# Modest effects of dietary supplements during the COVID-19 pandemic: insights from 445 850 users of the COVID-19 Symptom Study app

**DOI:** 10.1136/bmjnph-2021-000250

**Published:** 2021-04-19

**Authors:** Panayiotis Louca, Benjamin Murray, Kerstin Klaser, Mark S Graham, Mohsen Mazidi, Emily R Leeming, Ellen Thompson, Ruth Bowyer, David A Drew, Long H Nguyen, Jordi Merino, Maria Gomez, Olatz Mompeo, Ricardo Costeira, Carole H Sudre, Rachel Gibson, Claire J Steves, Jonathan Wolf, Paul W Franks, Sebastien Ourselin, Andrew T Chan, Sarah E Berry, Ana M Valdes, Philip C Calder, Tim D Spector, Cristina Menni

**Affiliations:** 1 Department of Twin Research and Genetic Epidemiology, King's College London, London, UK; 2 School of Biomedical Engineering & Imaging Sciences, King's College London, London, UK; 3 Clinical & Translational Epidemiology Unit, Massachusetts General Hospital, Boston, Massachusetts, USA; 4 Department of Clinical Sciences, Lund University, Lund, Sweden; 5 MRC Unit for Lifelong Health and Ageing at UCL, University College London, London, UK; 6 Department of Nutritional Sciences, King's College London, London, UK; 7 Zoe Global Limited, London, UK; 8 Division of Rheumatology, Orthopaedics and Dermatology, School of Medicine, University of Nottingham, Nottingham, Nottinghamshire, UK; 9 Human Development & Health, Faculty of Medicine, University of Southampton, Southampton, UK

**Keywords:** COVID-19, nutritional treatment

## Abstract

**Objectives:**

Dietary supplements may ameliorate SARS-CoV-2 infection, although scientific evidence to support such a role is lacking. We investigated whether users of the COVID-19 Symptom Study app who regularly took dietary supplements were less likely to test positive for SARS-CoV-2 infection.

**Design:**

App-based community survey.

**Setting:**

445 850 subscribers of an app that was launched to enable self-reported information related to SARS-CoV-2 infection for use in the general population in the UK (n=372 720), the USA (n=45 757) and Sweden (n=27 373).

**Main exposure:**

Self-reported regular dietary supplement usage (constant use during previous 3 months) in the first waves of the pandemic up to 31 July 2020.

**Main outcome measures:**

SARS-CoV-2 infection confirmed by viral RNA reverse transcriptase PCR test or serology test before 31 July 2020.

**Results:**

In 372 720 UK participants (175 652 supplement users and 197 068 non-users), those taking probiotics, omega-3 fatty acids, multivitamins or vitamin D had a lower risk of SARS-CoV-2 infection by 14% (95% CI (8% to 19%)), 12% (95% CI (8% to 16%)), 13% (95% CI (10% to 16%)) and 9% (95% CI (6% to 12%)), respectively, after adjusting for potential confounders. No effect was observed for those taking vitamin C, zinc or garlic supplements. On stratification by sex, age and body mass index (BMI), the protective associations in individuals taking probiotics, omega-3 fatty acids, multivitamins and vitamin D were observed in females across all ages and BMI groups, but were not seen in men. The same overall pattern of association was observed in both the US and Swedish cohorts.

**Conclusion:**

In women, we observed a modest but significant association between use of probiotics, omega-3 fatty acid, multivitamin or vitamin D supplements and lower risk of testing positive for SARS-CoV-2. We found no clear benefits for men nor any effect of vitamin C, garlic or zinc. Randomised controlled trials are required to confirm these observational findings before any therapeutic recommendations can be made.

## Introduction

A number of micronutrients, including vitamins C and D and zinc, have been shown to play key roles in supporting immune function^1 2^ and in reducing risk of respiratory infection.^2 3^ These nutrients can be obtained from the diet and are available as dietary supplements either alone or as part of multivitamin or multinutrient mixtures. There are many other dietary supplements available including, omega-3 fatty acids (‘fish oil’), probiotics and plant isolates like garlic.^4^ The use of specific dietary supplements in both prevention and acute treatment of infection with SARS-CoV-2 has been promoted by prominent medical entertainment personalities on television and social media since the beginning of the current coronavirus pandemic.^5^ The UK supplement market increased by 19.5% in the period leading up to the national ‘lockdown’ in early March 2020,^6^ with a 110% rise in sales of vitamin C and a 93% rise in sales of multivitamin supplements.^6^ Likewise, zinc supplement sales increased by 415% over the 7-day period ending 8 March, at the height of COVID-19 concern in the USA.^5^

A biologically plausible role exists for certain vitamins and minerals in immune pathways.^1^ For example, vitamin D has been suggested to reduce SARS-CoV-2 transmission by enhancing antiviral immunity and to reduce mortality by mitigating the cytokine storm linked with severe COVID-19.^7 8^ Moreover, zinc also supports the function of the immune system^9^ and may have specific antiviral effects.^10^ However, robust evidence to support a role for vitamins and minerals in preventing infection with SARS-CoV-2 is not available.^11^ Any such evidence would need to take into account factors such as socioeconomic status, ethnicity and occupational exposure to the virus as well as the requirement of a large sample size and a clear confirmation of infection.

By using data from the COVID-19 Symptom Study app,^12^ we tested the hypothesis that individuals taking dietary supplements, were at lower risk of testing positive for SARS-CoV-2, during the first wave of the pandemic. We initially examined whether supplement use was associated with SARS-CoV-2 infection among 372 720 UK participants who reported having been tested for SARS-CoV-2 using a reverse transcriptase-PCR (RT-PCR) or serology-based test. Next, we used data from 45 757 US and 27 373 Swedish (SE) app users who also reported tests for SARS-CoV-2 infection to replicate UK findings.

## Methods

### Study setting and participants

The COVID-19 Symptom Study app was developed by health data company Zoe Global with input from King’s College London, the Massachusetts General Hospital, Lund University, Sweden and Uppsala University, Sweden. In the UK, it was launched in English on Tuesday the 24 March 2020; in the USA in English and Spanish on Sunday the 29 March 2020 and in Sweden it was launched in Swedish on 29 April 2020 as previously described.^12 13^ Anyone over 18 years is able to sign up without any restriction. Individuals are also permitted to record information for dependencies under the age of 18. Here, we included individuals above 16 years of age (online supplemental figure S1). The app enabled self-reported information related to SARS-CoV-2 infection to be captured. On first use, the app recorded self-reported location, age and core health risk factors. With continued use, participants provided daily updates on symptoms, healthcare visits, SARS-CoV-2 test results and if they were self-quarantining or seeking healthcare, including the level of intervention and related outcomes. Individuals without apparent symptoms were also encouraged to use the app. Through direct updates, new or modified questions were added in real-time to capture data to test emerging hypotheses about COVID-19 symptoms and treatments. Here, we analysed data from the 31 July 2020 data dump.

10.1136/bmjnph-2021-000250.supp1 Supplementary data

### Assessment of exposure

Starting on the 2 June 2020, in the three countries, via the app, users were retrospectively asked if they had been taking supplements regularly (defined as: >3 times a week for at least 3 months) (online supplemental figure S1). Each user was able to fill in the supplements questionnaire only once (see online supplemental table S1 for list of questions). The questionnaire included use of probiotics, garlic, omega-3 fatty acids (‘fish oils’), multivitamins, vitamin D, vitamin C or zinc or no intake of any supplement. Supplement use was recorded as yes/no. For all the analyses, the control group consisted of individuals not taking any supplement.

10.1136/bmjnph-2021-000250.supp2 Supplementary data

### Ascertainment of outcomes

Participants were asked if they had been tested for COVID-19 using a RT-PCR or serology-based test and the results (none, negative, pending or positive). Our primary outcome was a report of a positive COVID-19 test between the time when they first reported on the COVID-19 Symptom Study app and 31 July 2020. Participants without a positive or negative test result were excluded (online supplemental figure S1).

### Ascertainment of covariates

Covariates including age, sex, body mass index (BMI), smoking, ethnicity, healthcare worker status and presence of comorbidities (ie, cancer, diabetes, eczema, heart disease, lung disease, kidney disease and hay fever) were self-reported via the app. The app also facilitated the index of multiple deprivation (IMD) to be generated from the relevant government websites of the UK,^14^ Scotland^15^ and Wales,^16^ with the most recent IMD available at the time of analysis used. The IMD was then categorised into quintiles within-population, where 1 is the least deprived and 5 is the most deprived. A subset of the UK app users (n=234 271) also completed a second retrospective questionnaire investigating diet quality at two time points. Here, we included data from the ‘peri-pandemic’ time point described as the previous month (from user access). The questionnaire included the validated Leeds Short Form Food Frequency Questionnaire (FFQ) developed by Cleghorn and collaborators and listed in the Nutritools (www.nutritools.org) library.^17 18^ We computed the validated Diet Quality Index, as previously described.^18^ Briefly, the Diet Quality Index was composed of fruit, vegetable, oily fish, fat and non-milk extrinsic sugar intakes reflecting five dietary components recognised as indicators of a healthy diet. Standard portion sizes were assigned to each food item on the FFQ.^18^

### Data sharing

Anonymised research data are shared with third parties via the centre for Health Data Research UK (HDRUK.ac.uk). US investigators are encouraged to coordinate data requests through the COronavirus Pandemic Epidemiology Consortium (www.monganinstitute.org/cope-consortium). Data updates can be found on https://covid.joinzoe.com https://covid.joinzoe.com.

### Statistical analysis

We studied 372 720 UK app users (aged 16–90 years) who self-reported information regarding regular dietary supplement usage and outcome of a COVID-19 test. Of these, 23 521 individuals tested positive for SARS-CoV-2 and 349 199 tested negative. Multivariate logistic regression adjusting for age, sex, BMI and health status at sign-up was applied to investigate the association between supplement use and testing positive for SARS-CoV-2. We then repeated the analyses (i) adjusting for age, sex, BMI, comorbidities (including type 2 diabetes, cancer, asthma, heart disease, eczema, hay fever, kidney disease and lung disease), IMD, smoking, ethnicity, health worker/carer status and diet quality and (ii) stratifying by sex, age group (<40, 40–60, >60 years) and BMI categories (normal weight, overweight, obese/morbidly obese).

Replication was conducted in two independent datasets including 45 757 US and 27 373 SE app users.

All p values presented were two-sided, with statistical significance determined by the Bonferroni-corrected threshold of significance (p=0.05/7=0.007). Statistical analysis was performed using Stata V.12 and ExeTera, a Python library developed at KCL to clean and process the raw dataset.^19^

### Patient and public involvement

No patients were directly involved in designing the research question or in conducting the research. No patients were asked for advice on interpretation or writing up the results. There are no plans to involve patients or relevant patient community in dissemination at this moment.

## Results

The demographic characteristics of the study population are presented in table 1. Briefly, our discovery cohort included 372 720 UK app users who reported having had an RT-PCR-based or serology test for SARS-CoV-2 and who completed the app-based dietary supplement questionnaire. The study sample was predominantly female (66.8%) and >50% were overweight (BMI (SD)=26.8 (5.6) kg/m^2^).

**Table 1:** Demographic characteristics of the study population

	UK (n=372 720)		USA (n=45 757)		SE (n=27 373)	
	Supplement users		Supplement users		Supplement users	
	Yes	No	Yes	No	Yes	No
N (%)	175 652 (47.1%)	197 068 (52.9%)	32 314 (70.6%)	13 443 (29.4%)	13 422 (49%)	13 951 (51%)
SARS-CoV-2 positive, *n (%)*	10 508 (6%)	13 013 (6.6%)	2002 (6.2%)	1211 (9%)	1806 (13.5%)	2206 (15.8%)
Females, *n (%)*	123 462 (70.3%)	125 651 (63.8%)	22 817 (70.6%)	8210 (61.1%)	9694 (72.2%)	9088 (65.1%)
White, *n (%)*	163 479 (93.1%)	188 030 (95.4%)	28 143 (87.1%)	11 757 (87.5%)	13 411 (99.9%)	13 943 (99.9%)
Current smoker, *n (%)*	6004 (3.4%)	10 773 (5.5%)	1252 (3.9%)	924 (6.9%)	735 (5.5%)	795 (5.7%)
	*Mean (SD*)	*Mean (SD*)	*Mean (SD*)	*Mean (SD*)	*Mean (SD*)	*Mean (SD*)
Age, years	49.57 (14.2)	46.26 (14.4)	56.24 (15.2)	47.8 (16.0)	49.0 (13.0)	46.63 (12.9)
BMI, kg/m^2^	26.59 (5.6)	27.04 (5.7)	27.27 (5.9)	27.21 (6)	26.1 (4.8)	26 (4.7)
IMD, median (IQR)	7 (5)	6 (5)	–	–	–	–
DQI, median (IQR)	11 (3)	11 (3)	–	–	–	–
	*N* (*%*)	*N* (*%*)	*N* (*%*)	*N* (*%*)	*N* (*%*)	*N* (*%*)
Omega-3	39 263 (22.4%)	–	8663 (26.8%)	–	3039 (22.6%)	–
Probiotics	20 449 (11.6%)	–	7268 (22.5%)	–	1715 (12.8%)	–
Garlic	7235 (4.1%)	–	1137 (3.5%)	–	941 (7%)	–
Multivitamins	77 034 (43.9%)	–	18 843 (58.3%)	–	5496 (41%)	–
Vitamin D	86 190 (49.1%)	–	19 444 (60.2%)	–	6722 (50.1%)	–
Vitamin C	46 755 (26.6%)	–	10 136 (31.4%)	–	4045 (30.1%)	–
Zinc	21 776 (12.4%)	–	4330 (13.4%)	–	2394 (17.8%)	–
Type 2 diabetes	5047 (2.9%)	4842 (2.5%)	1787 (5.5%)	437 (3.3%)	331 (2.5%%)	251 (1.8%%)
Cancer	2178 (1.2%)	1902 (1%)	977 (3%)	217 (1.6%)	157 (1.2%)	130 (0.9%)
Asthma	27 146 (15.5%)	26 020 (13.2%)	5015 (15.5%)	1788 (13.3%)	2039 (15.2%)	1673 (12%)
Heart disease	5708 (3.3%)	5278 (2.7%)	2388 (7.4%)	590 (4.4%)	654 (4.9%)	592 (4.2%)
Eczema	22 896 (13%)	23 454 (11.9%)	3423 (10.6%)	1346 (10%)	1768 (13.2%)	1779 (12.8%)
Hay fever	79 024 (45%)	80 114 (40.7%)	17 000 (52.6%)	6256 (46.5%)	5497 (41%)	5256 (37.7%)
Kidney disease	1702 (1%)	1542 (0.8%)	630 (2%)	143 (1.1%)	117 (0.9%)	102 (0.7%)
Lung disease	24 651 (14%)	22 924 (11.6%)	4331 (13.4%)	1329 (9.9%)	1894 (14.1%)	1488 (10.1%)

Self-reported comorbidities were not asked at sign-up and therefore not reported by all subjects.

BMI, body mass index; DQI, Diet Quality Score; IMD, index of multiple deprivation; SE, Sweden.

As shown in table 1, out of the 372 720 UK app users, 175 652 (47%) self-reported using supplements regularly since the beginning of the pandemic, while 197 068 self-reported they were not taking any supplement. This is in line with the UK general population supplement usage as reported in the National Diet and Nutrition Survey.^4 20^

**Figure 1:**
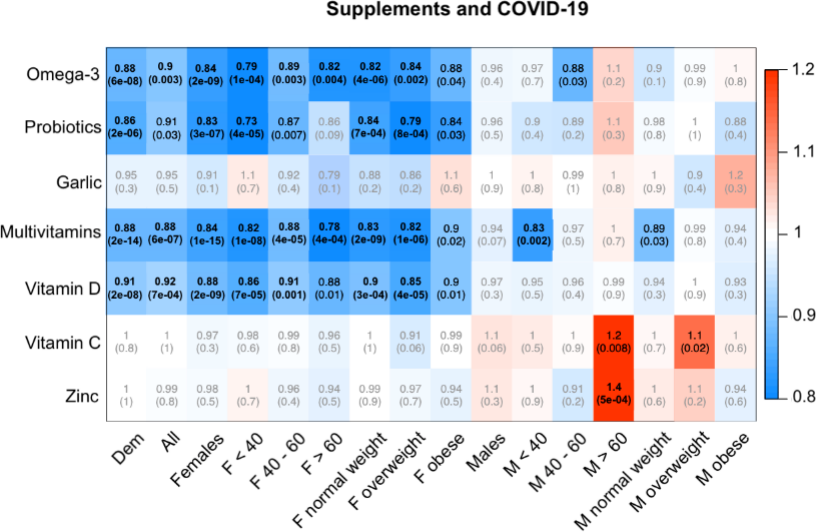
Associations between testing positive for SARS-CoV-2 and self-reported use of supplements in UK app users. Each cell of the matrix displays the OR of the association between use of a type of supplement and testing positive with the corresponding p value in parentheses. The table is colour coded according to the OR, with blue denoting a reduced risk and red denoting an increased risk of testing positive. Bold entries are statistically significant after accounting for multiple testing using Bonferroni correction. Dem, adjusted for age, sex, body mass ndex (BMI) and health status at sign up; All, adjusted for Dem, index of multiple deprivation, ethnicity, comorbidities (type 2 diabetes, cancer, asthma, heart disease, eczema, hay fever, kidney disease and lung disease), smoking, diet quality; stratified analyses are adjusted for age, (BMI) and health status at sign up as appropriate.

In the UK cohort, users regularly supplementing their diet (i) with multivitamins had a lower risk of testing positive for SARS-CoV-2 by 13% (OR (95% CI)=0.87 (0.84 to 0.90), p=1.62×10^−14^), (ii) with vitamin D had a lower risk by 9% (OR (95% CI)=0.91 (0.88 to 0.94), p=2.07×10^−8^); (iii) with probiotics had a lower risk by 14% OR (95% CI)=0.86 (0.81 to 0.92), p=1.99×10^−6^) and (iv) with omega-3 fatty acids had a lower risk by 12% (OR (95% CI)=0.88 (0.84 to 0.92), p=5.8×10^−8^), after adjusting for age, sex, BMI, sign-up health status and multiple testing (figure 1). There were no significant associations in those supplementing with zinc, vitamin C or garlic (figure 1). Crude results are presented in online supplemental table S2. To account for a potential healthy user bias, we did a sensitivity analysis further adjusting for ethnicity, comorbidities, smoking, IMD and health worker/carer status, results remained consistent with the previous analysis (figure 1), although the effect of probiotics appeared weaker. We also adjusted for the Diet Quality Index. In line with the literature,^21^ we found diet quality and supplements usage to be positively correlated (r=0.07, p<0.0001), supporting that people whose diet are closer to the recommended one are more likely to take supplements compared with those whose diet is far from the recommendation. However, when we adjust for diet quality, results remain consistent, suggesting that the effect of supplement use is independent from the effect of diet quality (figure 1).

10.1136/bmjnph-2021-000250.supp3 Supplementary data

Next, we ran the analyses stratifying by sex, age group and BMI categories and we detected sexual dimorphism in the association between supplement use and testing positive for SARS-CoV-2 (figure 1). Females taking probiotics, omega-3 fatty acids, multivitamins and vitamin D had a lower risk of infection across all age groups and BMI categories (OR (95% CI) ranging from 0.73 (0.63 to 0.85) for probiotics in women <40 years of age to 0.91 (0.86 to 0.96) for vitamin D in women aged between 40 and 60 years). No protective association was observed in males overall. However, in post hoc subgroup analyses, men aged <40 years or normal weight and taking multivitamins and men aged 40–60 years and taking omega-3 fatty acids were less likely to be infected (figure 1). In contrast, there was a positive association in men aged >60 years taking zinc (1.4 (1.16 to1.69), p=4.92×10^−4^) or vitamin C supplements (1.22 (1.05 to 1.41), p=8.1×10^−3^) (figure 1) for testing positive for SARS-CoV-2.

**Figure 2:**
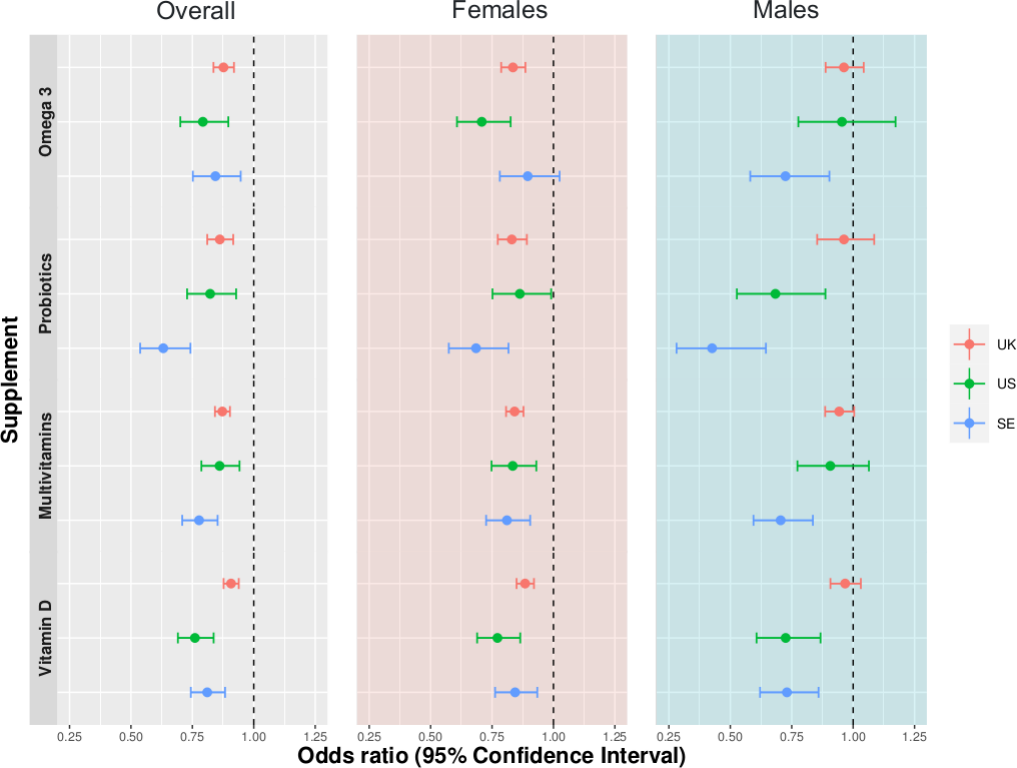
ORs and 95% CIs for the associations between testing positive for SARS-CoV-2 and self-reported use of supplements in three cohorts (n=372 720 UK, n=45 575 USA and n=27 373 SE). Overall sample analyses are adjusted for age, sex, body mass index (BMI) and health status at sign up. Analyses according to sex are adjusted for age, BMI and health status at sign up.

To replicate significant findings from the UK cohort, we next used data from the 45 757 US and 27 373 SE app users. Cohorts were similar, in that they were also predominantly female (USA: 67.8%, SE: 68.6%) and a greater proportion were overweight (BMI (SD), USA: 27.3 (5.9) kg/m^2^, SE: 26 (4.7) kg/m^2^). Overall UK findings were mirrored in both cohorts (figure 2). However, findings by gender varied different in different cohorts (figure 2).

Associations in females were replicated in the US cohort, but omega-3 supplement use was not associated with testing positive in Swedish females (figure 2). US males using probiotics or vitamin D had a decreased risk of a positive test for SARS-CoV-2 (figure 2), while Swedish men taking probiotics, omega-3 fatty acids, multivitamins or vitamin D had a decreased risk of infection (figure 2).

## Discussion

In the largest observational study on SARS-CoV-2 infection and dietary supplement use to date on over 400 000 app users from three different countries, we show a significant association between users of omega-3 fatty acid, probiotic, multivitamin or vitamin D supplements and lower risk of testing positive for infection with SARS-CoV-2. However, our stratified analysis in the tested group shows a strong sexual dimorphism with the consistent protective effect present only in females, at least in the UK, and for some supplements in the USA and SE. This association has several potential explanations including: (i) biological explanations include discordant immune systems between sexes that could respond differently to supplements.^22^ Indeed, a sexual dimorphism in nutrient metabolism has been previously reported, with females having a more robust immune response than men.^23^ Moreover, females typically possess a more resilient immune system than males with higher numbers of circulating B cells when matched for age, BMI and clinical parameters,^24^ as well as a slower age-related decline in circulating T cells and B cells.^24^ It is therefore plausible that supplements could better support the immune system of females than males, although the lack of consistency between countries is problematic; (ii) differences in body weight and body composition between males and females meaning that supplement dosing on a per body weight basis may be higher in females^25^; (iii) residual confounding due to sex differences in health-related behaviours, including COVID-19.^26^ Polling reveals that a greater percentage of females versus males are anxious for the health of themselves or their family and therefore are more precautionary, cancelling plans and staying home more often.^26^ Females who purchase vitamins may also be more health conscious than males, such as having greater use of wearing face masks and hand-washing.^27–29^ Indeed, in our data, we found that women tended to wear masks more often than males (44% of women report wearing a mask at least some of the time when outside, compared with 36% of men, p<0.001).

### Vitamin D

A potential antimicrobial role of vitamin D in infections dates back almost a century,^30^ with several mechanistic study supporting a positive physiological role.^3^ Immune cells express the vitamin D receptor and some can synthesise the active form of vitamin D. Vitamin D influences the function of antigen-presenting cells, T cells and B cells.^31^ It also promotes production of cathelicidin, a microbicidal component of the innate immune system.^32^ The overlap between risk factors for vitamin D deficiency and risk of severe COVID-19, such as obesity, age and ethnicity, gives some plausibility to a protective role of vitamin D.^30^ A meta-analysis of 39 randomised controlled trials reported that vitamin D reduces the risk of respiratory infections by around 11%, but there was considerable heterogeneity.^33^ However, a Mendelian randomisation study suggests that genetic levels of vitamin D are not associated with COVID-19 susceptibility,^34^ in line with recent results from the UKBiobank.^35^

In our data, we find a modest protective effect for infection, with a 9% reduction in risk of testing positive for SARS-CoV-2 in the overall UK cohort, 24% in the US cohort and 19% in the SE group.

#### Multivitamins

Multivitamin supplements typically include multiple vitamins and multiple minerals including trace elements^36^; many of these have antioxidant properties and roles in supporting the immune system.^1 2^ Specific micronutrient deficiencies, including zinc, selenium, vitamin A, vitamin D and vitamin E, have been shown to be detrimental during viral infections.^37–39^ Although some randomised controlled trials have shown that multivitamin supplements reduce the risk of respiratory infections,^40^ a recent review argues that this evidence is weak and unclear.^41^ Here, we provide evidence to support a modest protective effect in those taking multivitamin supplements similar to vitamin D with a 13% reduction in risk of testing positive for SARS-CoV-2 in the overall UK cohort, 12% in the US cohort and 22% in the SE cohort.

#### Omega-3 fatty acids

Omega-3 fatty acids can influence antigen-presenting cell, T-cell and B-cell function, although their effects on these cell types in humans is not consistently reported. However, they are clearly demonstrated to be anti-inflammatory^42^ and to be converted to specialised proresolving mediators such as resolvins, protectins and maresins.^43^ Whether this is a mechanism by which they reduce risk of testing positive for SARS-CoV-2 is not clear. Nevertheless, here we provide evidence to support a protective effect in omega-3 fatty acid supplements users with a 12% reduction in risk of testing positive for SARS-CoV-2 in the overall UK cohort, 21% in the US cohort and 16% in the SE cohort. Although, in the UK, stratified analysis shows this effect is largely driven by females, and only significant in one male stratum.

#### Probiotics

Probiotics modify the host’s gut microbiota and may generate antiviral metabolites, and they interact with the host’s gut-associated immune system.^44^ This can result in improved immunity, including enhanced responses to the seasonal influenza vaccine.^45^ Mechanistic studies support a gut-lung axis,^46^ whereby immune effects of microbiota at the gut level can be transferred to the lung, most likely through movement of immune cells. This could explain why some probiotic organisms reduce risk^47–49^ and severity^50^ of respiratory tract infections. Here, we provide evidence to suggest that people taking probiotics supplements are modestly protected with a 14% reduction in risk of testing positive for SARS-CoV-2 in the overall UK cohort, 18% in the US cohort and 37% in the SE cohort. However, effects of probiotics are strain and species specific and we have no information of which probiotics or their quality were being used by participants in this study. Moreover, when we adjusted for other covariates including diet, the effect of probiotics was weaker suggesting that probiotic use may be confounded by a healthy diet.

#### Zinc, vitamin C and garlic

We saw no protective effects of zinc, garlic or vitamin C. Both zinc and vitamin C have been previously suggested to support the immune system and to prevent respiratory infections.^1 2 51^ Although, their efficacy and evidence base has been questioned and a meta-analysis of vitamin C showed no preventive benefit but a reduction in severity and a modest reduction in symptom duration.^52 53^

Strengths of our study include its large sample size, the confirmation of SARS-CoV-2 through a RT-PCR-based or serology-based test, and the replication of the key findings from the UK cohort in two other cohorts, one in the US cohort and the other in SE cohort. Furthermore, we had information on diet quality. Indeed, consistent with the literature,^21^ in our data we found a positive correlation between diet quality and supplements usage suggesting that people with a healthier diet were more likely to take supplements. However, we were able to adjust for diet quality and showed that the effect of supplements is independent from the effect of diet quality.

Our study also has a number of limitations. First, we used self-reported data which can introduce information bias, including misclassification, or effect bias exposure if participants started taking supplements after developing symptoms. There is also a possibility of participants having COVID-19 symptoms (and likely having COVID-19) but not having had any test because of low testing capacity^54^ and then started taking supplements. We believe this is possible, although would have reduced any real effect. To help mitigate potential spurious associations, we adjusted for self-reported health status at sign-up. Second, participants using the app were a self-selected group and may not be fully representative of the general population. Indeed, our study population is predominantly female, although supplement usage and BMI are in line with data for the UK general population.^4 20^ This may cause collider bias, that is, both use of supplements and having COVID-19 might influence the probability of an individual participating in the COVID-19 Symptom Study, and this selection effect could introduce an apparent association between supplement use and COVID-19 status.^55^ Third, the SARS-CoV-2 infection diagnosis was mainly based on the RT-PCR test that has <100% sensitivity (true positive rate).^56 57^ Fourth, participants might have been taking supplements in addition to the seven we asked them about, and we were not able to account for multiple sources of vitamins/minerals. Fifth, malnutrition is a significant risk factor for a severe form of COVID-19 infection,^58^ however we had information on diet, we were unable to infer vitamin/mineral deficiencies. Sixth, we do not know the exact intakes of the ingredients within the supplements used by the participants, nor we had any information regarding dosage and hence we were unable to link supplements dosage or intakes from multiple sources with disease outcome. Furthermore, within the useful timeframe, the app has not captured information on behaviours such as washing hands, use of disinfectants or social distancing and only a proportion of participants replied to the questions on use of masks. We also were only able to adjust for a crude, area-based, measure of deprivation rather than individually based measures of socioeconomic background, such as educational attainment, occupation and income; thus, residual confounding by socioeconomic status is possible. Finally, this is observational data captured during a specific timeframe, and our study design does not allow an inference of causality.

In conclusion, our data show that women taking multivitamins, omega-3 fatty acids, vitamin D or probiotics have a slightly lower risk of SARS-CoV-2 infection in the UK, US and SE cohorts, but no effect in those taking zinc, vitamin C or garlic. Given the interest in supplements during the pandemic, large randomised controlled trials of selected supplements testing their protective effects, and also possible adverse effects, on disease severity are required before any evidence-based recommendations can be made. We eagerly await the result of ongoing trials, including of vitamin D, omega-3 fatty acids and probiotics and COVID-19 risk.^59^

**What this paper adds**

**What is already known on this topic**

- Dietary supplements have been shown to play key roles in supporting immune function, but the extent to which specific supplements are associated with reduced risk of SARS-CoV-2 infection is not known.

**What this study adds**

- Individuals taking multivitamins, omega-3 fatty acids, probiotics or vitamin D were less likely to be tested positive for SARS-CoV-2 in three large independent cohorts of app users.
- There was a significant protective association for vitamin D, omega-3 fatty acids, probiotics and multivitamins in female users across all ages and body mass index categories within the largest (UK) cohort; yet, there was no association in male users of this cohort.
- Vitamin C, zinc and garlic supplements had no association with risk for SARS-CoV-2.
- There is a need for randomised controlled trials of selected supplements.

## Data Availability

Data are available on reasonable request. Anonymised research data are shared with third parties via the centre for Health Data Research UK (HDRUK.ac.uk). US investigators are encouraged to coordinate data requests through the COPE Consortium (www.monganinstitute.org/cope-consortium). Data updates can be found on https://covid.joinzoe.com.
