# Supplementary figures and images for: Modest effects of dietary supplements during the COVID-19 pandemic: insights from 445 850 users of the COVID-19 Symptom Study app
Source: BMJ Nutr Prev Health. 2021 Apr 19;4(1):149–57. doi: 10.1136/bmjnph-2021-000250 (PMC8061565; doi:10.1136/bmjnph-2021-000250)

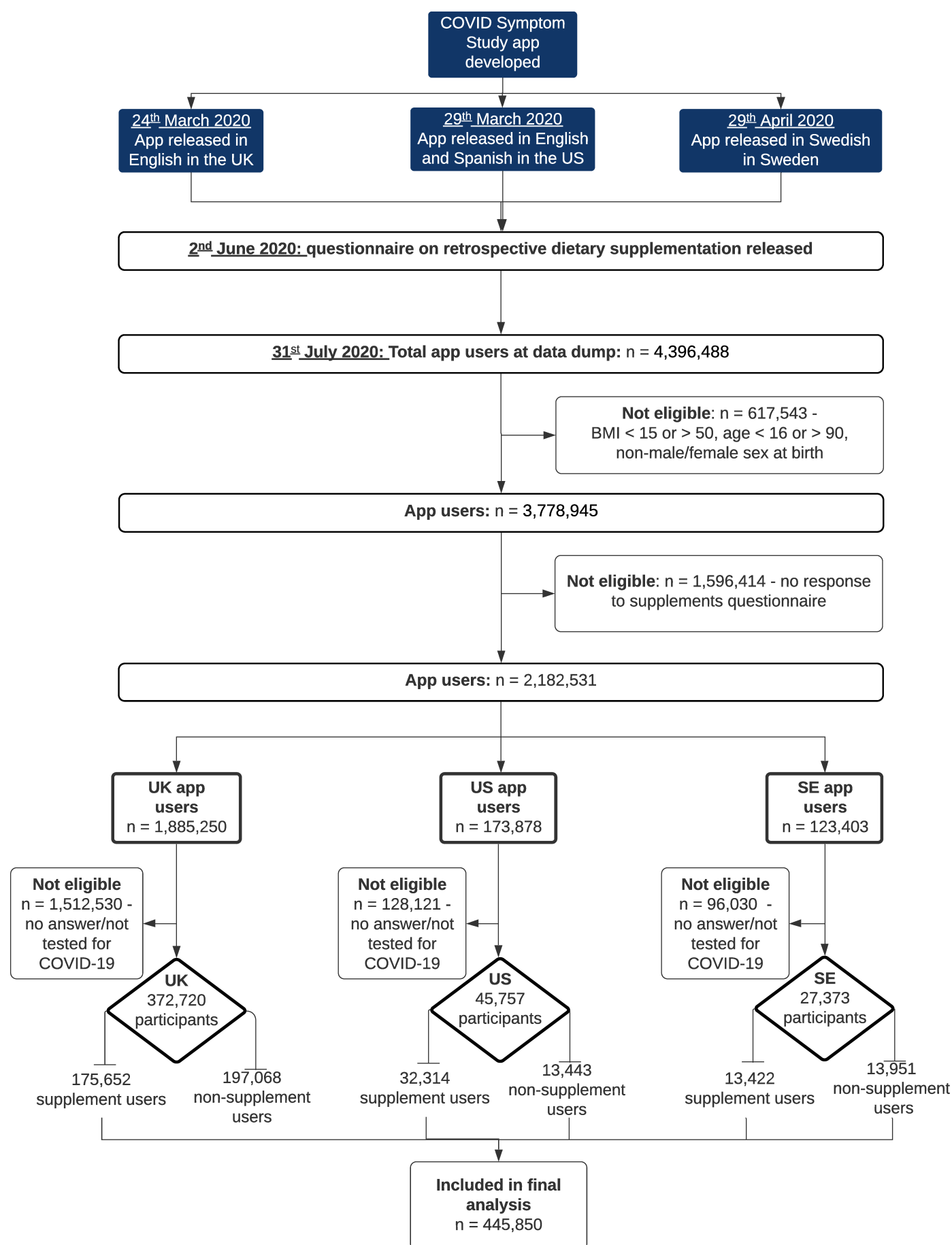

Supplement: Supplementary data [file bmjnph-2021-000250supp001.pdf]
