# Supplementary material for: Modest effects of dietary supplements during the COVID-19 pandemic: insights from 445 850 users of the COVID-19 Symptom Study app
Source: BMJ Nutr Prev Health. 2021 Apr 19:bmjnph-2021-000250. doi: 10.1136/bmjnph-2021-000250 (PMC8061565; doi:10.1136/bmjnph-2021-000250)
Supplement: Supplementary file 1 [file bmjnph-2021-000250supp001.pdf]

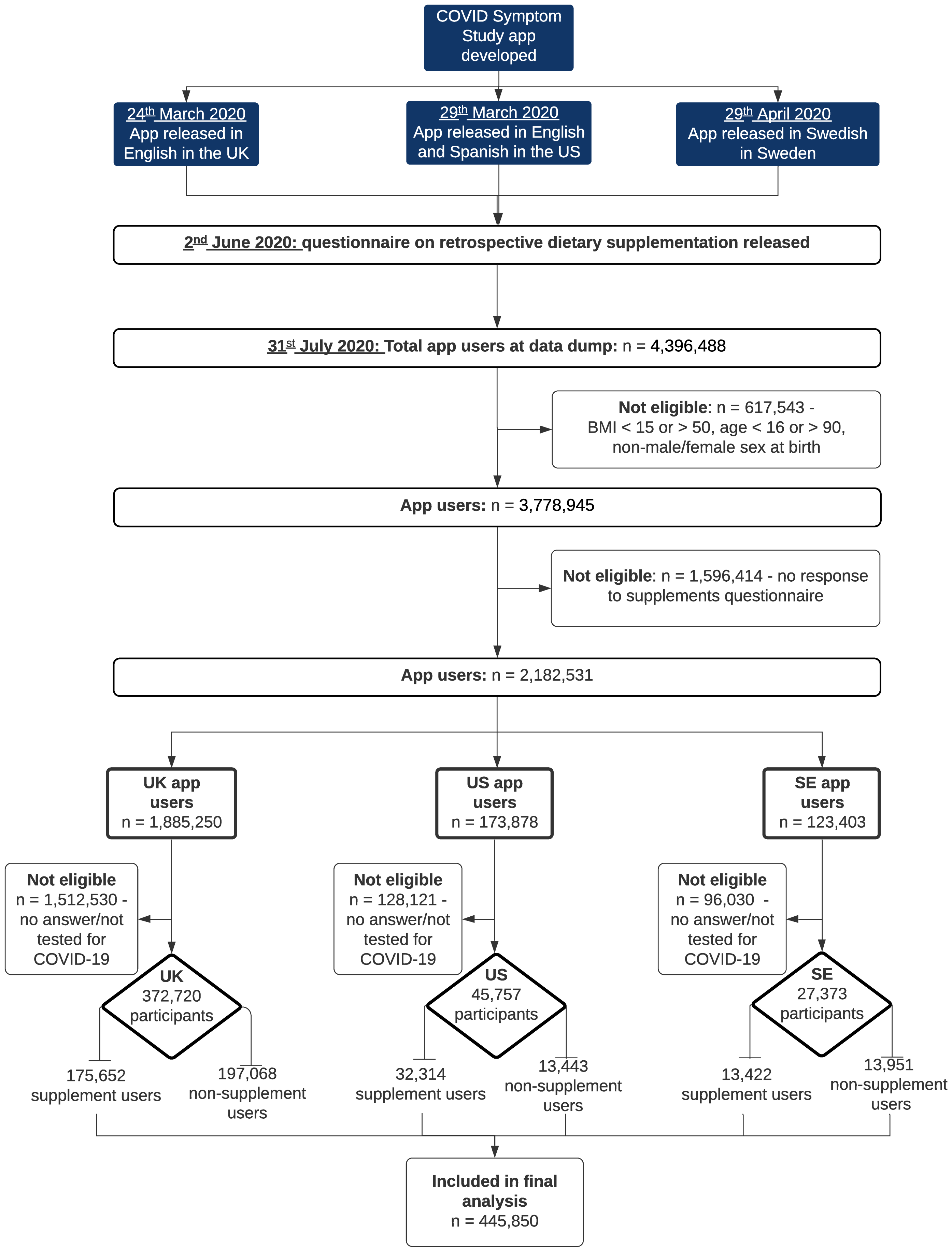

COVID Symptom Study app developed
24th March 2020 App released in English in the UK
29th March 2020 App released in English and Spanish in the US
29th April 2020 App released in Swedish in Sweden
2nd June 2020: questionnaire on retrospective dietary supplementation released
31st July 2020: Total app users at data dump: n = 4,396,488
Not eligible: n = 617,543 - BMI < 15 or > 50, age < 16 or > 90, non-male/female sex at birth
App users: n = 3,778,945
Not eligible: n = 1,596,414 - no response to supplements questionnaire
App users: n = 2,182,531
UK app users n = 1,885,250
US app users n = 173,878
SE app users n = 123,403
Not eligible n = 1,512,530 - no answer/not tested for COVID-19
Not eligible n = 128,121 - no answer/not tested for COVID-19
Not eligible n = 96,030 - no answer/not tested for COVID-19
UK 372,720 participants
US 45,757 participants
SE 27,373 participants
175,652 supplement users
197,068 non-supplement users
32,314 supplement users
13,443 non-supplement users
13,422 supplement users
13,951 non-supplement users
Included in final analysis n = 445,850
