# Supplementary material for: Modest effects of dietary supplements during the COVID-19 pandemic: insights from 445 850 users of the COVID-19 Symptom Study app
Source: BMJ Nutr Prev Health. 2021 Apr 19:bmjnph-2021-000250. doi: 10.1136/bmjnph-2021-000250 (PMC8061565; doi:10.1136/bmjnph-2021-000250)
Supplement: Supplementary file 2 [file bmjnph-2021-000250supp002.pdf]

Table S1. List of questions on supplements usage

Have you been taking any vitamins or other supplements regularly for more than 3 months? Regularly means more than 3 times a week on average. Select all that apply.
**- No**
**- Vitamin C**
**- Vitamin D**
**- Omega-3 or Fish Oil**
**- Zinc**
**- Garlic**
**- Probiotics**
**- Multi-vitamins and minerals**
**- Other, please specify**
**- Prefer not to say**
