# Supplementary material for: Modest effects of dietary supplements during the COVID-19 pandemic: insights from 445 850 users of the COVID-19 Symptom Study app
Source: BMJ Nutr Prev Health. 2021 Apr 19:bmjnph-2021-000250. doi: 10.1136/bmjnph-2021-000250 (PMC8061565; doi:10.1136/bmjnph-2021-000250)
Supplement: Supplementary file 3 [file bmjnph-2021-000250supp003.pdf]

Table S2. Associations between testing positive for SARS-CoV-2 and self-reported use of supplements in UK, US and SE app users. Crude OR[95%CI] are reported.

	UK		US		SE	
**Supplement**	**OR[95%CI]**	**P**	**OR[95%CI]**	**P**	**OR[95%CI]**	**P**
**Omega-3**	0.82[0.79;0.85]	3.57E-22	0.56[0.5;0.62]	1.28E-24	0.86[0.76;0.96]	8.96E-03
**Probiotics**	0.88[0.83;0.92]	1.32E-07	0.69[0.62;0.77]	9.66E-11	0.67[0.56;0.77]	4.53E-07
**Garlic**	1.03[0.95;1.11]	5.25E-01	0.98[0.78;1.19]	8.87E-01	0.88[0.72;1.05]	2.00E-01
**Multivitamins**	0.88[0.85;0.91]	3.42E-17	0.69[0.63;0.74]	5.94E-19	0.8[0.73;0.88]	1.97E-06
**Vitamin D**	0.88[0.85;0.9]	1.19E-19	0.58[0.53;0.63]	2.46E-35	0.83[0.76;0.9]	1.66E-05
**Vitamin C**	1.02[0.99;1.06]	1.97E-01	0.81[0.73;0.89]	1.63E-05	0.91[0.82;0.99]	4.86E-02
**Zinc**	1.06[1.02;1.11]	8.16E-03	0.92[0.81;1.03]	1.92E-01	0.91[0.79;1.02]	1.12E-01
